# Non-technical skills in the out-of-hospital cardiac arrest: is it time for a pit stop?

**DOI:** 10.29045/14784726.2019.03.3.4.45

**Published:** 2019-03-01

**Authors:** Stef Cormack

**Affiliations:** Coventry University

## Abstract

**Aims:**

To ascertain an understanding and identify if there are barriers to the use of non-technical skills (NTS) in an out-of-hospital cardiac arrest (OHCA) by student paramedics.

**Methods:**

An initial literature review was completed using online databases (Medline, PubMed, AMED, PsycINFO, PsycARTICLES, CINAHL) and MeSH terms (prehospital, paramedic, ambulance, EMS, non-technical skills, soft skills, crew resource management, team resource management, human factors). A mixed methods study using an adapted questionnaire (using existing, validated NTS questions) was applied to a convenience sample of student paramedics. Twenty-five Likert scaled closed-ended questions and three open-ended questions were asked, followed by a focus group. Pearson’s chi-squared and correlation statistical tests were used to examine differences in questionnaire answers, with thematic analysis used to analyse open-ended questions and focus group answers.

**Results:**

The literature review revealed a paucity of evidence for NTS in OHCA but did identify key NTS in the in-hospital environment: communication, leadership and situational awareness. Barriers to NTS included a lack of understanding of roles, a lack of clinical ability and poor planning. Fifty student paramedics (29 Trust employed and 21 direct entry) participated in the questionnaire, 31 male and 19 female. Most were aged 18–39 and had less than five years’ operational experience, with a mean of 8.5 OHCA attended per year (SD 8.55). Overall there was no statistical difference between students, however direct entry students were more likely to receive a briefing before attending an OHCA (p = 0.03) while Trust students were more likely to delegate during an OHCA (p = 0.001). There was an association that more staff attending an OHCA increased the use of checklists (p = 0.001), improved awareness (p = 0.006) and delegation of tasks (p = 0.01). There was a correlation between the switching of roles (p = 0.02) and increased task delegation (p = 0.05) the more OHCAs attended. There was no statistical difference found for gender or length of service. Five key themes were identified from open-question answers: teamwork (leadership and followership), situational awareness, communication, emotional intelligence and decision making. The focus group identified that students felt that leadership was not as important as teamwork or effective communication and there was confusion over what situational awareness was.

**Conclusion:**

Student paramedics are aware of NTS in the OHCA but there is a limited understanding of situational awareness and how to overcome barriers to improving NTS. The development of a behavioural marker tool to improve NTS in an OHCA would be beneficial.

